# Physical Exam and Evaluation of the Unstable Shoulder

**DOI:** 10.2174/1874325001711010946

**Published:** 2017-08-31

**Authors:** María Valencia Mora, Miguel Ángel Ruiz Ibán, Jorge Diaz Heredia, Juan Carlos Gutiérrez-Gómez, Raquel Ruiz Diaz, Mikel Aramberri, Carlos Cobiella

**Affiliations:** 1 Hospital Universitario Fundación Jiménez Díaz, Madrid, Spain; 2 Hospital Universitario Ramón y Cajal, Madrid, Spain; 3 University College of London, London, United Kingdom

**Keywords:** Hyperlaxity, shoulder Dislocation, instability, physical Examination, sports

## Abstract

**Background:**

The clinical evaluation of the patient with shoulder instability can be challenging. The pathological spectrum ranges from the straightforward “recurrent anterior dislocation” patient to the overhead athlete with a painful shoulder but not clear instability episodes. Advances in shoulder arthroscopy and imaging have helped in understanding the anatomy and physiopathology of the symptoms. The aim of this general article is to summarize the main examination manoeuvres that could be included in an overall approach to a patient with a suspicion of instability.

**Material and Methods:**

In order to achieve the above-mentioned objective, a thorough review of the literature has been performed. Data regarding sensibility and specificity of each test have been included as well as a detailed description of the indications to perform them. Also, the most frequent and recent variations of these diagnostic tests are included.

**Results:**

Laxity and instability should be considered separately. For anterior instability, a combination of apprehension, relocation and release tests provide great specificity. On the other hand, multidirectional or posterior instability can be difficult to diagnose especially when the main complain is pain.

**Conclusion:**

A detailed interview and clinical examination of the patient are mandatory in order to identify a shoulder instability problem. Range of motion of both shoulders, clicking of catching sensations as well as pain, should be considered together with dislocation and subluxation episodes. Specific instability and hyperlaxity tests should be also performed to obtain an accurate diagnosis.

## 1 INTRODUCTION

The shoulder is the most commonly dislocated joint in the body and is especially vulnerable in sporting activities with overhead involvement [1, 2]. The concepts of shoulder laxity and instability have changed in the last decades, and so has done physical examination [3-5]. Our knowledge of the physiopathology of shoulder instability has evolved together with magnetic resonance imaging and arthroscopic techniques. This has lead to the definition of a vast spectrum of instability types with associated lesions affecting capsulolabral, ligamentous and osseous structures [6]. For the clinician, detecting instability of the shoulder may be considered an easy problem, as anterior traumatic glenohumeral dislocations account for more than 95% of the shoulder dislocations [6]. However, other glenohumeral instability types such as non-traumatic, posterior or multidirectional instability can be harder to diagnose when the most prominent symptom is pain [7]. Moreover, provocative tests that are helpful in order to identify one instability type can be misleading for the other types.

### 1.1 History

The consultation should start with an exhaustive history including personal and sporting background. For either an isolated episode or recurrent instability, age at the time of the first episode as well as mechanism of injury should be recorded [5]. Whether or not it was reduced at the hospital or it resolved spontaneously, as well as any neurological complication, are also important details that can give an idea of the severity of the first episode. In chronic instability cases, one should include the number of dislocations, activities during which it happens and type of reduction needed.

### 1.2 Physical Exam

Physical examination should always be performed bilaterally to provide comparison. It should include visual inspection, palpation, active and passive range of motion and motor and sensory testing. Then, a general shoulder examination can be performed, including specific tests selected according to clinical suspicion. The diagnosis of occult instability should be always considered in young athletes with a painful shoulder [8].

The aim of this review is to analyze the characteristics of physical examination in different laxity and instability patterns.

## 2 LAXITY EXAMINATION

When performing an examination of the shoulder the clinician should be able to differentiate between laxity and instability. The amount of movement allowed in each joint is defined as laxity of the joint. In the shoulder, the bony anatomy of the proximal humerus and glenoid, the negative intra-articular pressure, the ligaments, the rotator cuff tendons and the compressive forces of the muscles determine stability [9, 10]. Laxity is mainly determined by looseness of the passive stabilizers. When there is excessive laxity it can be limited to this joint or it can be generalized. The Beighton Hypermobility Score is a simple system to quantify joint laxity [11]. It uses a 9-point system where the higher the score, the greater the laxity. The threshold for joint hiperlaxity in a young adult is ranged from 4 to 6. It evaluates dorsiflexion of the fifth finger and the thumb, hyperextension of knees and elbows and ability to forward flex the trunk.

When referring to the shoulder, the “gold standard” to evaluate shoulder laxity would be examination under anesthesia, because it eliminates the action of the active stabilizers of the joint [12]. What can be considered the normal degree of laxity in the shoulder is unknown, but it has been demonstrated that it decreases with age, as the passive stabilizers tighten [11] and is increased in patients with anterior glenohumeral instability [13, 14]. Shoulder laxity examination should be performed in the same arm position and applying the same amount of force in order to avoid inter-observer variability. Although it should not cause pain, patients with previous dislocations might be difficult to explore.

Most of the tests performed to evaluate laxity are based on measurement of humeral head displacement on the glenoid in any direction [1]. A cadaveric study by Sauers *et al.* [15] showed that the mean displacement of the humeral head in the glenoid was anteriorly 11.8mm, posteriorly 8.6 mm and inferiorly 20.2 mm.

Several methods have been described in order to quantify the amount of anterior or posterior displacement. Hawkins *et al.* [16] recommended a classification based on millimeters. Grade 0 is defined as no translation, grade I represents a translation smaller than 1 cm, grade II is considered when there is a moderate translation of 1 to 2 cm (or up to the glenoid rim) and grade III equals severe translation of more than 2cm or over the glenoid rim. An alternative method consists in measuring the percentage of humeral head diameter that is translated. Thus, the humeral head is said to move up to the glenoid rim (grade I), over the glenoid rim but less than 50% of the head diameter (grade II) or over the glenoid rim more than 50% of the head diameter (grade III). However, Harryman *et al.* [17] do not recommend this method because of the low estimated accuracy. The third method, most used in clinical practice, is based on describing what the examiner feels [18]. It was also described by Hawkins and includes four grades; Grade 0: no translation, grade I: translation to the glenoid rim, grade II: translation of the head over the glenoid rim and grade III, when the head stays out of the joint after removing the examiner hands.

### 2.1 Load and Shift Test

The aim of this test is to demonstrate the amount of antero-posterior translation of the humeral head on the glenoid. Silliman and Hawkins first described it in 1991 [18] and it can be performed with the patient sitting or supine [19]. With the patient sitting, the examiner stabilizes the scapula by placing one hand over the top of the shoulder while the other is placed on the proximal arm. It is important to load the humeral head into the glenoid before starting anterior or posterior displacement. This test has not been validated using biomechanical techniques. Tzannes and Murrell found a sensitivity of 50% for anterior instability and 14% for posterior instability while the specificity was of 100% for both of them [19].

### 2.2 Drawer Test

Gerber and Ganz described anterior and posterior drawer tests in 1984 [20]. Examination is performed with the patient supine and the examiner to the side.

The posterior drawer test is performed by holding the patient´s wrist [20] or forearm [1] with one hand and placing the other hand over the patient´s shoulder so that the thumb is in the front and the fingers in the back. The thumb should be placed over the humeral head while applying a posteriorly directed force. The arm should be forwardly flexed at the same time in order to allow the head to subluxate posteriorly. The displacement is measured as explained before. Saha *et al.* [21] described the “zero unpacked position” (arm elevated 45º to 60º) as the position in which shoulder has the most mobility. These authors recommend performing the posterior drawer test in this position to obtain more reliable results.

**Fig. (1):**
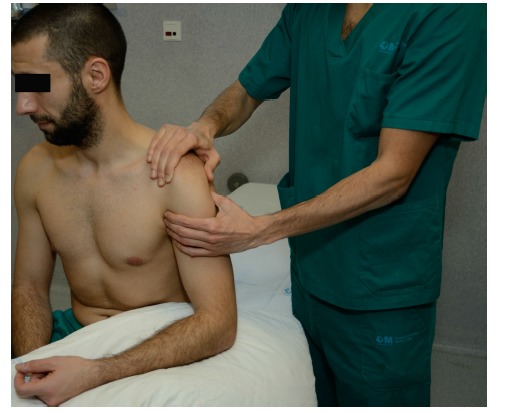
**Drawer test**. One hand stabilizes the scapula with the thumb in the coracoid and the fingers in the scapular spine. The other hand creates an anteriorly directed force to provoke and anterior translation of the humeral head.

For the anterior drawer test the examiner has to control scapular rotation. The arm is abducted 80º to 120º, flexed 0º to 20º and held in 0º to 30º of external rotation [20]. In this case, one hand stabilizes the scapula with the thumb in the coracoid and the fingers in the scapular spine. The other hand creates an anteriorly directed force to provoke and anterior translation of the humeral head. McFarland *et al.* [1] described another technique with the arm in 40º of abduction and slight internal rotation. One hand is placed on the arm and the other in the wrist, while applying an axial load to the shoulder. In this position, the authors recommend the force to be applied in the whole arm and not only in the humeral head (Fig. **1**).

It has been described that grade II displacement could be considered normal among young athletic populations and that asymmetry of the shoulders should not be used as a criterion for diagnosis of shoulder laxity [22].

### 2.3 Sulcus Test

**Fig. (2):**
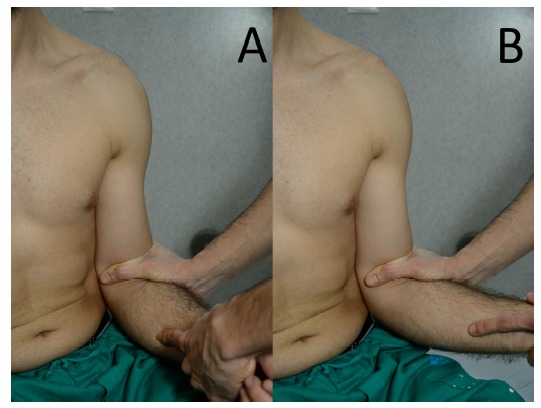
**Sulcus sign.** The arm is held first in neutral rotation (**a**) and secondly in external rotation (**b**). The elbow is grasped and pulled inferiorly. If the test is positive, a sulcus appears in the subacromial region as the humeral head translates in the inferior direction.

Neer and Foster [23] described the sulcus test in 1980 in the context of a multidirectional instability study and it has remained as a way to examine inferior laxity. Later biomechanical investigation has demonstrated that it evaluates the superior glenohumeral ligament [24, 25]. This examination can be performed with the patient standing, sitting or supine. McFarland *et al.* have suggested that more reliable data can be obtained with the patient sitting and the arm by the side [1]. The elbow is grasped and pulled inferiorly (Fig. **2**). If the test is positive, a sulcus appears in the subacromial region as the humeral head translates in the inferior direction [19]. It has to be repeated twice, first with the arm in neutral rotation and secondly with the arm in external rotation. Inferior translation should be the same in both positions. In the case that we find an increased translation with the arm in external rotation a lesion of the rotator interval should be considered [25, 26] (Fig. **2**).

The amount of displacement can be measured in centimeters. Typically, grade I would imply a displacement of less than 1.5 cm, grade II between 1.5 and 2 cm and grade III more than 2 cm. McFarland [1] has suggested grouping these values as low (grade I) and high (grades II and III). Tzannes and Murrell [27] found that a positive sulcus sign of more than 2 cm had a sensitivity of 28% and a specificity of 97%. However, other authors have demonstrated a wide range of inferior laxity in asymptomatic patients, suggesting that there is no absolute degree of inferior translation that defines inferior instability [1].

### 2.4 Hyperabduction Test

**Fig. (3):**
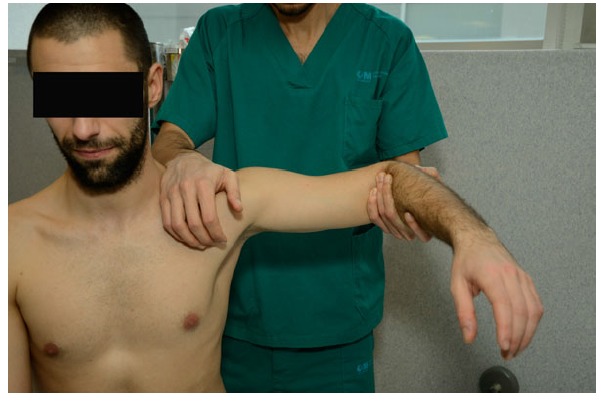
**The *Hyperabduction* test** by Gagey is performed as shown. Passive abduction is performed until the scapula starts moving. It should be performed bilaterally to obtain a comparison.

This test was reported by Dr. O.J Gagey and Dr. N. Gagey in 2001 to assess laxity in the inferior glenohumeral complex [28]. It is performed with the examiner standing behind the patient using one arm to stabilize the scapula (Fig. **3**). The patient´s arm is abducted until the scapula is felt to start moving. The test is positive if the range of passive abduction is over 105º. The authors reported a 15% of patients in which passive abduction was limited by apprehension what could somehow contribute to the diagnosis of instability [28]. At present, this test has evolved to the “comparative hyperabduction test” in which both shoulders are examined and three items should be noted in order to consider the result positive. First, it has to reproduce patient´s deep pain; Secondly, it has to be asymmetrical when compared to the contralateral side (>20º) and third; a soft end point should not be felt [8] (Fig. **3**).

### 2.5 Posterior Laxity Examination

Various tests have been described in order to examine posterior shoulder laxity. The push-pull test [29] and the Protzman test [30] could be considered modifications of the posterior load and shift test. Clarnette and Miniaci [31] also described a posterior subluxation test performed in internal rotation, adduction and flexion that has not yet been validated.

## 3 INSTABILITY: PROVOCATIVE MANEUVERS

### Anterior Instability Examination

Anterior glenohumeral instability is the most frequent problem encountered [5]. Thus, most of the tests have been described in order to diagnose it. Same as in laxity examination, the testing methods and what is considered a positive test are still controversial [32].

#### 3.1 The Apprehension Sign/ Augmentation test

**Fig. (4):**
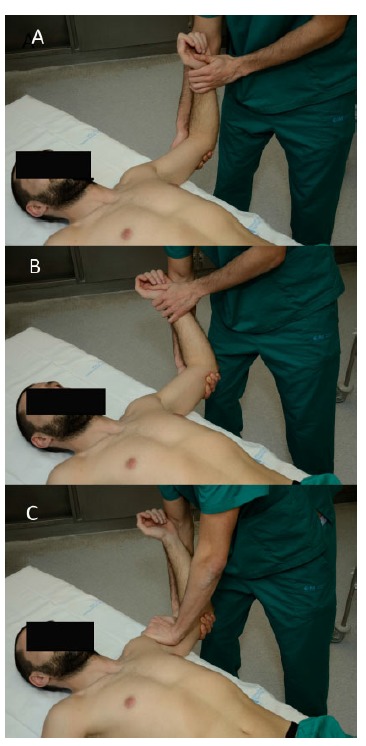
**Anterior apprehension test and relocation test.** The shoulder is held in a position of 90º of abduction with the elbow at 90º of flexion and external rotation is applied from there until the patient feels pain or becomes apprehensive (**a** & **b**). Then, a posteriorly directed force is applied on the anterior aspect of the shoulder (**c**). The examiner should notice if the patient experiences relief of apprehension or just relief of pain in order to consider it positive.

Since Rowe and Zarins [33] first described this test for anterior shoulder instability many variations have been suggested. It can be performed either standing or supine. In the standing position, the examiner places the arm in external rotation and abduction of 90º with one hand while the other applies a gentle pressure anteriorly. Farber *et al.* [3] reported more reliable results when including this test in the general examination routine, holding both arms by the forearm and exploring both at the same time without exerting any pressure on the shoulder. Another way to perform the apprehension test is with the patient in supine position, placed on the side of the table keeping the scapula supported (Fig. **4**). The shoulder is held in a position of 90º of abduction with the elbow at 90º of flexion and the examiner´s knee supporting the elbow to prevent extension of the shoulder [32]. From there, external rotation is applied until the patient feels pain or becomes apprehensive. Apprehension used as a criterion has demonstrated to achieve better inter-observer agreement whereas pain might overestimate the number of positive results [27, 32, 34]. When pain is felt, the examiner should ask the patient to localize it in the posterior, anterior or lateral aspect of the shoulder. The specificity of this test has been reported as 98.9% with low sensitivity of 52.8% [32].

Several variations of this test have been described. In the “augmentation test” [35], the “crank test” [35] and the “fulcrum test” [29] an anterior force is applied in the position of maximum apprehension.

#### 3.2 Relocation Test

Frank Jobe described this test in 1989 [36] as an examination to be performed in overhead athletes in order to detect pain arising from the rotator cuff in what he called “secondary impingement”. He believed that the anterior band of the inferior glenohumeral complex was stretched in throwers, which meant increased external rotation. This increased range of motion allowed the humeral head, and consequently the rotator cuff, to impinge upon the acromion. Later on, with Walch´s description of “internal impingement” the meaning of the examination changed [37]. It is currently performed after completion of the apprehension test, usually in patients with a history of previous dislocations. The arm is placed in 90º of shoulder abduction and 90º of elbow flexion and external rotation is performed until the patient reports pain and/or apprehension. Then, a posteriorly directed force is applied on the anterior aspect of the shoulder. The examiner should notice if the patient experiences relief of apprehension or just relief of pain to distinguish between frank instability and occult instability with symptoms of internal impingement (Fig. **4**). It has been reported that relocation test can cause pain in 82% of patients with rotator cuff disease, 63% with posterior instability and 80% with acromioclavicular disorders and could be useful in the diagnosis of posterior SLAP lesions [34, 38]. Therefore, the area where the pain is elicited should be recorded to avoid a misleading diagnosis. Lo *et al.* [32] reported a sensitivity of only 45.83% and a specificity of 54.35%. Similarly to the apprehension test, specificity raised to 100% when relief of apprehension was considered the criteria for a positive test, rather than pain relief.

#### 3.3 The Release and Surprise Tests

The release test can be performed as a part of the relocation test. Once in the position of maximum abduction and external rotation, the humeral head is stabilized with a posterior force as in the relocation test. This should allow us to slightly increase external rotation while exerting this posterior force. Should we suddenly remove the hand from the front of the shoulder, the patient will feel increasing pain or apprehension as the humeral head moves forward. The surprise test only differs in that external rotation should not be increased [32]. Both of them can cause extreme discomfort to the patient or even a dislocation and should therefore be performed carefully.

### 3.4 Posterior Instability Examination

Posterior instability is much less common than anterior instability, comprising between 2% and 10% of all reported instability cases [39]. This means it can be missed in the absence of a clear traumatic dislocation episode such as in sporting activities, a seizure episode or an electrical shock that gives rise to suspicion. The mechanism of injury or situations that reproduce the symptoms should be recorded and might orientate the clinician to this type of instability, as they usually coexist with other shoulder problems such as impingement or labral tears [39]. In young athletes, as mentioned before, laxity should not be mistaken for instability. Although examination at first glance could seem benign, Pollock *et al.* [40] described that patients are commonly tender to palpation along the posterior joint line, possibly from synovitis provoked by multiple episodes of instability. Von Raebox *et al.* have also reported the presence of a small skin dimple or tether over the posteromedial deltoid of both shoulders in patients with posterior positional dislocation [41].

Many provocative maneuvers have been described. The posterior apprehension sign was described as a test in which the arm was pushed posteriorly while being held in a position of flexion, adduction and internal rotation [29]. However, not all the patients with confirmed posterior instability feel the symptoms in the same position [21]. Later, O´Driscoll and Evans described the “posterior apprehension test for pain” that consisted in reproducing a Kennedy-Hawkins impingement sign. In the cases in which pain was elicited, a subacromial injection was performed to rule out glenohumeral symptoms [42].

**Fig. (5):**
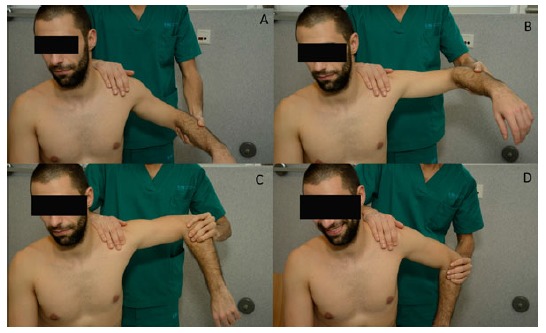
**Jerk test.** With the patient seated, the shoulder is stabilized with one hand. With the other hand, the patient grasps the elbow and abducts the arm to 90º while internally rotating and applying an axial load directed to the shoulder (**a** & **b**). Keeping the axial load, the arm is adducted until a sudden clunk or jerk appears (**c** & **d**).

**Fig. (6):**
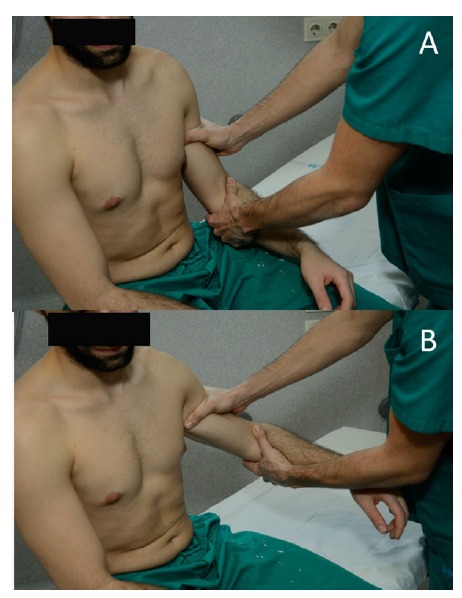
**Kim test.** (**a**) While the patient seated, the examiner grabs the elbow with one hand and the biceps area with the other (**b**). The arm is then passively elevated while applying a posterior force to the upper arm and an axial load to the elbow. A positive result would mean pain or subluxation.

The Jerk test was described by Matsen *et al.* [29] The arm is brought into flexion, adduction and internal rotation with the elbow flexed as well. Then, the examiner applies an axial force along the humerus to induce a posterior subluxation (Fig. **5**). The Kim test is performed with the patient sitting (Fig. **6**). The examiner holds the elbow with one hand and keeps the other over the patient’s biceps. Then, the arm is then passively flexed 45º while applying a downward and posterior force to the upper arm and an axial load to the elbow [43]. When performed together, they have been reported to provide a sensitivity of 97% for detecting postero-inferior labral tears [43].

## CONCLUSION

A thorough interview and a general bilateral examination should always be performed in order to orientate the provocation tests and our diagnosis. Laxity should be always explored and should not be confused with instability, particularly in young patients. For anterior glenohumeral instability, a combination of apprehension, relocation and release test can provide great specificity. For posterior instability, although many provocation tests have been described, diagnosis still remains challenging.
